# Roles of extracellular vesicles in glioblastoma: foes, friends and informers

**DOI:** 10.3389/fonc.2023.1291177

**Published:** 2023-11-24

**Authors:** Taral R. Lunavat, Lisa Nieland, Anne B. Vrijmoet, Ayrton Zargani-Piccardi, Youssef Samaha, Koen Breyne, Xandra O. Breakefield

**Affiliations:** ^1^ Molecular Neurogenetics Unit, Massachusetts General Hospital and Harvard Medical School, Charlestown, MA, United States; ^2^ Department of Biomedicine, University of Bergen, Bergen, Norway; ^3^ Department of Neurosurgery, Leiden University Medical Center, Leiden, RC, Netherlands

**Keywords:** glioblastoma, tumor microenvironement, extracellular vesicles, pro-tumorigenic, immunosuppressive biomarkers

## Abstract

Glioblastoma (GB) tumors are one of the most insidious cancers which take over the brain and defy therapy. Over time and in response to treatment the tumor and the brain cells in the tumor microenvironment (TME) undergo many genetic/epigenetic driven changes in their phenotypes and this is reflected in the cellular contents within the extracellular vesicles (EVs) they produce. With the result that some EVs try to subdue the tumor (friends of the brain), while others participate in the glioblastoma takeover (foes of the brain) in a dynamic and ever changing process. Monitoring the contents of these EVs in biofluids can inform decisions based on GB status to guide therapeutic intervention. This review covers primarily recent research describing the different cell types in the brain, as well as the tumor cells, which participate in this EV deluge. This includes EVs produced by the tumor which manipulate the transcriptome of normal cells in their environment in support of tumor growth (foes), as well as responses of normal cells which try to restrict tumor growth and invasion, including traveling to cervical lymph nodes to present tumor neo-antigens to dendritic cells (DCs). In addition EVs released by tumors into biofluids can report on the status of living tumor cells via their cargo and thus serving as biomarkers. However, EVs released by tumor cells and their influence on normal cells in the tumor microenvironment is a major factor in immune suppression and coercion of normal brain cells to join the GB “band wagon”. Efforts are being made to deploy EVs as therapeutic vehicles for drugs and small inhibitory RNAs. Increasing knowledge about EVs in the TME is being utilized to track tumor progression and response to therapy and even to weaponize EVs to fight the tumor.

## Introduction

From the human perspective, any factors that support progression of glioblastomas (GBs) are considered foes and any that hinder their growth or support therapeutic intervention are friends. GB is an extremely fast growing and almost always a lethal malignancy arising presumably from neural precursor or glial cells in the brain (1). The lethality of the GB can be attributed in part to the usually advanced stage at the time of diagnosis and the many modes of resistance to treatment. Extracellular vesicles (EVs) are a stealthy means of communication within the brain that can transfer components of one cell to other cells, thereby altering their physiologic state. EVs are secreted by all cell-types in the tumor microenvironment (TME), including the tumor cells themselves, and have unique and conflicting roles in this fight for survival. These vesicles are membrane enclosed, retaining the orientation of the membrane of the cells from which they are derived. Typically, 50-200 nm in diameter they contain cargo from the source cell including protein, RNA, DNA, lipids and sugars (2, 3). Tumor-derived EVs can be considered as packets of directives to instruct other cells on how to respond to the tumor, inform on the status of the tumor, and potentially be manipulated to contribute to therapeutic intervention. They have an important role, working in conjunction with secreted factors and cell-to-cell contact in changing the phenotype of normal cells in the TME, controlling immune responses to the tumor and regulating the rate of tumor cell proliferation and invasion into the brain.

**Figure 1:**
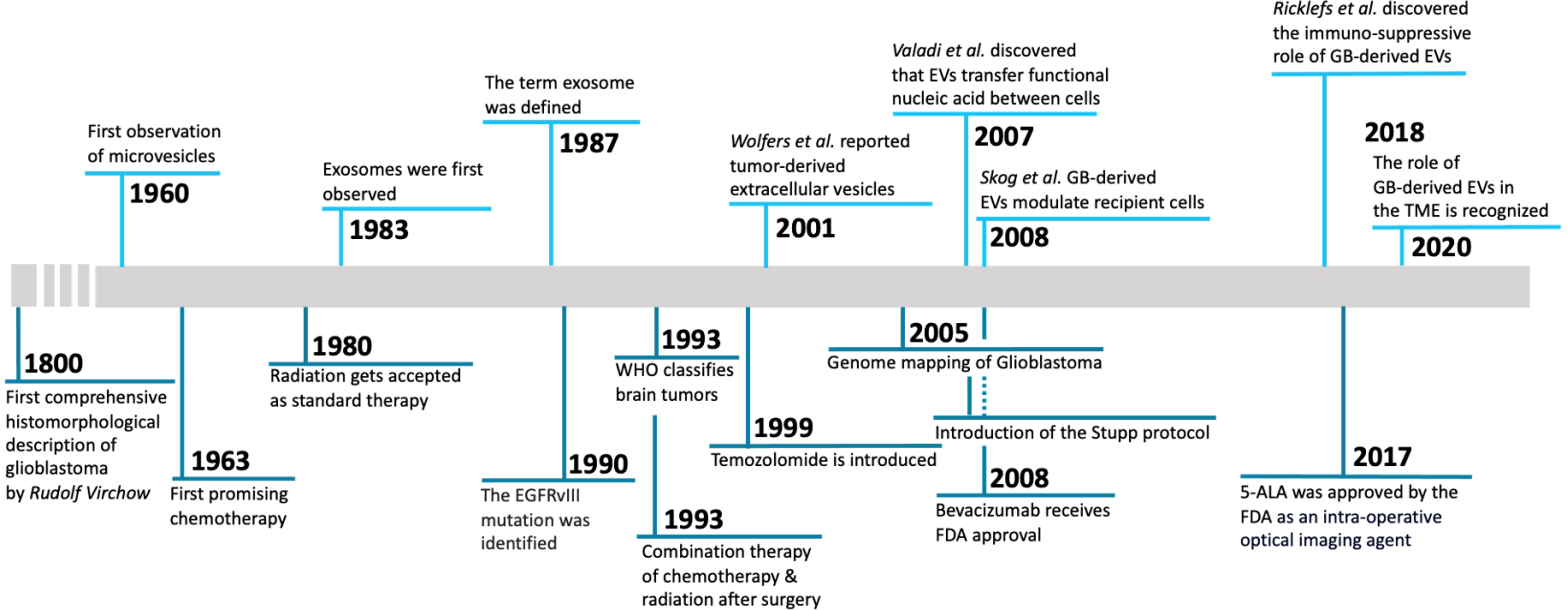
Timeline of milestone discoveries regarding GB-derived EVs. 1800, First comprehensive histomorphological description of glioblastoma by Rudolf Virchow (11). 1960, First observation of microvesicles (12). 1963, First promising chemotherapy (13). 1980, Radiation is accepted as standard therapy (14). 1983, Exosomes were first observed (15). 1987, The term exosome was defined (16). 1990, The EGFRvIII mutation was identified (17). 1993, WHO classifies brain tumors (18). 1993, Combination therapy of chemotherapy & radiation after surgery (19). 1999, Temozolomide is introduced (20). 2001, Report of tumor-derived extracellular vesicles (21). 2005, Genome mapping of glioblastoma (22). 2005, Introduction of the Stupp protocol (23). 2007, Discovery that extracellular vesicles transfer functional nucleic acid between cells (24). 2008, EV RNA as blood biomarker for GB and GB-derived extracellular-vesicles modulate recipient cells (25). 2008, Bevacizumab receives FDA approval (26). 2017, 5-ALA approved by the FDA as an intra-operative optical imaging agent (27). 2018, Discover of the immuno-suppressive role of glioblastoma-derived extracellular vesicles (28). 2020, The role of glioblastoma-derived extracellular vesicles in the tumor microenvironment is recognized (29).

This review will focus on advances in understanding these dynamic interactions in research articles over the past five-or-so years. We also recommend a few other reviews which have provided insight into this ongoing dialogue, although there is still much to be discovered (4–6). Other relevant reviews include protumorigenic mechanisms in GB (7); interactions of tumor EVs in radiotherapy (8), therapeutic vesicles for GB (9) and EVs as biomarkers for GB (10). A timeline (**Figure 1**) of important publications related to EVs in GB is provided.

## Foe activity – EVs promote tumor progression

### GB-derived EVs change the phenotype of surrounding brain cells in support of tumor growth

**Figure 2:**
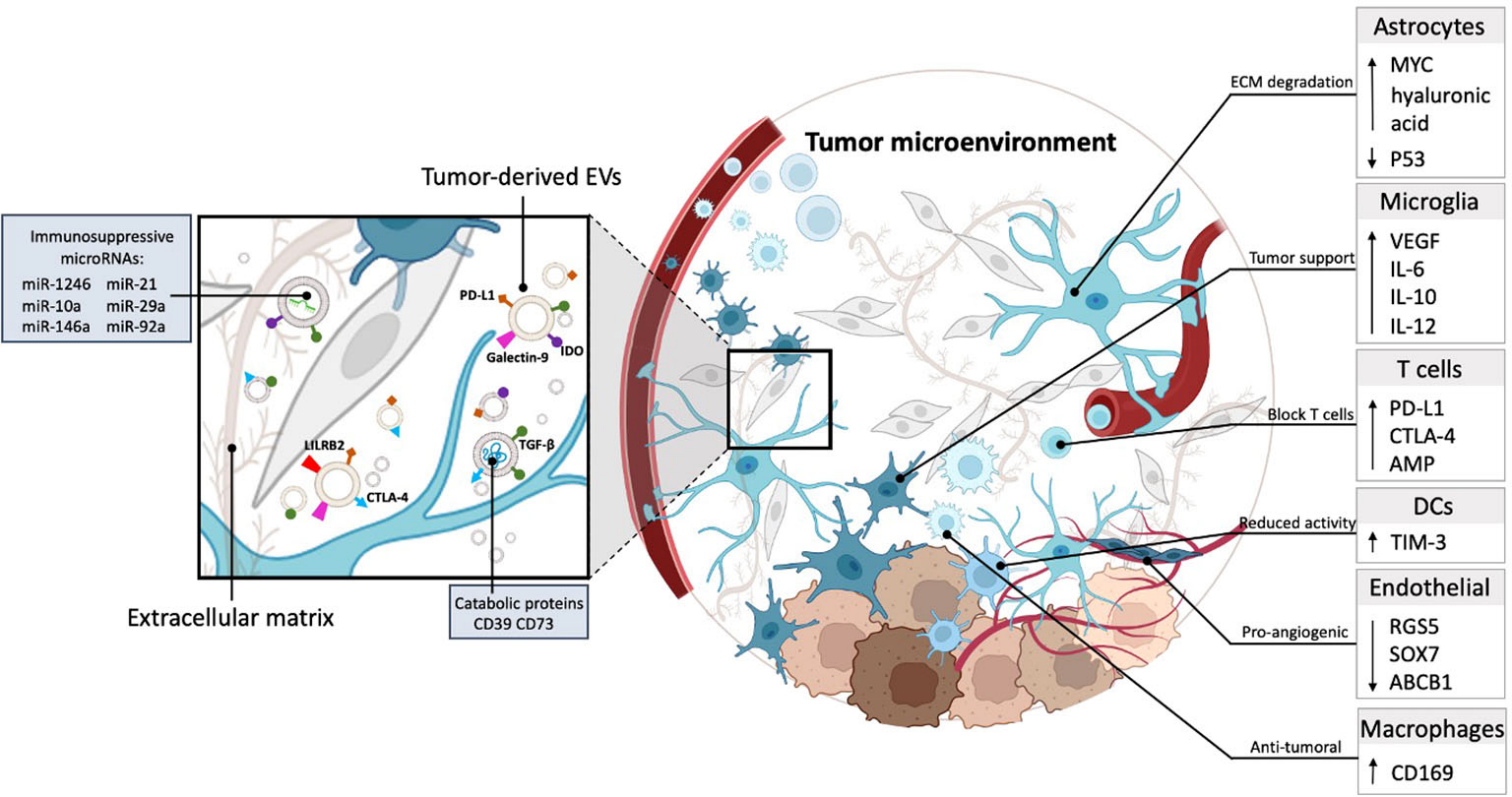
GB-derived EVs are capable of hijacking healthy brain cells to promote tumor growth. Tumor-derived EVs carry catabolic proteins and express immunosuppressive molecules, including PD-L1, TGF-β, IDO, and galectin 9. Moreover, they contain immunosuppressive miRNAs (miR-1246, miR-10a, miR-21, miR-29a, miR-92a). These EVs operate within the ECM and are taken up by cells in the TME, contributing to immune evasion and other tumor-promoting processes (left). The TME consists of many cell types, including astrocytes, microglia, T cells, DCs, endothelial cells, and macrophages. Astrocytes contribute to the degradation of the ECM within the TME. Microglia cells within the TME promote tumor growth by increasing the production of pro-inflammatory and endothelial factors, including VEGF, IL-6, IL-12, and IL-10. T cells are hindered within the TME due to the increased expression of immune checkpoint proteins, including PD-L1, CTLA-4, and AMP. This blockade restricts the functionality of T cells within the TME. DCs in the TME potentially reduce tumor cell functionality by increasing the expression of TIM-3, which may contribute to impairing tumor cell function. Endothelial cells in the TME promote angiogenesis, by downregulating RGS5, SOX7, and ABCB1, thereby creating a pro-angiogenic environment. A unique group of macrophages identifiable by the CD169 marker contributes to the establishment of an anti-tumor surrounding within GB.

GBs are known for their heterogeneity between patients and even at the genome/phenotype level within a single tumor (30–32), as well as the complexity of the TME, which comprises various cell-types, including tumor cells, neurons, microglia, astrocytes, macrophages, endothelial cells and immune cells (4). Glioma cells affect almost all cell types in the TME, and recruit non-tumor cells to support glioma expansion, such as monocytes from the bloodstream (33) and microglia from other areas of the brain (34). Multiple studies have shown that GB cells are capable of hijacking healthy brain cells to promote tumor growth through “instructions” mediated in part by EV cargo (35, 36) (**Figure 2**). EVs regulate gene expression by surface signaling and depositing their cargo into cells in their proximity and at even more distant sites, and are also released into the cerebral spinal fluid (CSF) and blood (37). For example, EVs mediate crosstalk between GB cells and astrocytes, the latter being the most abundant glial cells in the brain (38). GB cells secrete EVs that alter normal astrocytes, which are intended to protect healthy brain tissue, and turn them into highly reactive GB-associated astrocytes via activation of MYC and inhibition of p53 pathways (39). These GB-associated astrocytes transition into a tumor-promoting phenotype characterized by secretion of pro-inflammatory molecules, such as interleukin (IL)-6 (39), increase in their migrational capacity with enhanced cytokine production through signaling pathways, such as nuclear factor kappa B (NF-κB) and transforming growth factor-β (TGF-β) (40). In addition, through JNK signaling, high levels of CD147 secreted by GB lead to matrix metalloproteinase-mediated degradation of the extracellular matrix (ECM) supporting tumor growth and invasiveness (41).

In addition, GB-derived EVs promote vascularization via reprogramming of endothelial cells, a main component of the perivascular niche – the microenvironment around a blood vessel (42). RNA-sequencing (RNA-seq) analysis has identified candidate microRNAs (mi-RNAs), including miR-9 which mediates post-transcriptional downregulation of angiostatic genes, including *RGS5*, *SOX7*, and *ABCB1* and could explain the failure of anti-angiogenic therapy using anti-vascular endothelial growth factor (VEGF) strategies (43). RNAseq analysis further revealed that microglia, the innate immune cells of the brain which have taken up tumor-derived EVs downregulate genes that are involved in sensing tumor cells and generating an immune reponse to tumor neo-antigens, and actually end up supporting tumor growth (34).

GB-derived EVs also promote proliferation and migration of neuronal progenitor cells through the PI3K-Akt-mTOR pathway (44) and can potentially participate in transformation of these stem-like cells, such that they become tumor-like and may participate in support of tumor recurrence (45). Moreover, EVs play a role in resistance to therapy through their function as decoys of antibody-based therapy, or as drug efflux transporters, as elaborated in a recent review (5). Additionally, GB-derived EVs are involved in radiation-resistance through specific mi-RNA cargosuch as mir320e, miR520f-3p, miR363-3p, miR144-4p, miR16-5p, miR495-3p, miR23a-3p, and miR155-5p which target the PTEN pathway (46).

In conclusion, current evidence points towards a pro-tumorigenic role for GB-derived EVs in modulating the TME, reprogramming healthy brain cells towards a more tumor supportive state and protecting tumor cells from therapy. Although studies have shown that glioma-derived EVs affect neighboring cells in the brain, the many mechanisms by which GB-EVs regulate the TME and affect current therapeutic strategies needs to be further elucidated. One major player - miR-21 is high in GB cells and knocking out miR-21 results in reduced tumor growth (47). GB EVs also have high miR-21 and when transferred to microglia results in changes in their transcriptome which support tumor growth (48). Further studies are crucial to understand glioma-derived EV communication and its interplay with healthy cells of the brain, which could potentially open new therapeutic avenues.

### GB-derived EVs suppress the immune response to tumor antigens

Overall, GBs effectively counter anti-tumor immunity essential for a positive immunotherapy outcome (49). As well-recognized intercellular mediators, GB EVs incorporate immune attenuating molecules, such as checkpoint inhibitor proteins - programmed death ligand-1 (PD-L1;(28), cytotoxic T-lymphocyte associated protein 4 (CTLA-4;(50), and immunosuppressive cytokines, such as TGF-β (29). GB EVs also contain small immunosuppressive mi-RNA species such as miR-1246, miR-10a, miR-21, miR-29a, and miR-92a, which serve to generate an “immunosuppressive halo” around tumor cells (51). The goal of these regulatory signals transported by GB-derived EVs is to manipulate oncogenic cells associated with the tumor for example microglia, myeloid-derived suppressor cells, and dendritic cells (DCs), while blocking potential anti-tumor activity in the TME by interfering with the recruitment from the periphery and activity of immune cells, including CD4+ effector T cells and CD8+ effector T cells (52).

EVs mimic immunosuppressive signals that act through direct contact with immune target cells. GB-derived EVs are enriched with membrane-associated PD-L1 interacting with PD-1+ tumor-reactive T cells to impair their proliferation and stimulation (28). In addition to PD-L1, other immune checkpoint proteins, such as CTLA-4, can be exposed to GB-derived EVs and act to suppress natural killer (NK) cell and CD4+ T cell activation (50). The same study showed that CD39 and CD73 are also transported by these EVs. These catabolic proteins convert ADP/ATP into AMP or adenosine, leading to blockage of clonal expansion and homing of T cells by interacting with the adenosine receptor, A2AR (53–55). Another inhibitory molecule transferred by GB-EVs known to modulate T cells is leukocyte immunoglobulin-like receptor subfamily 2 (LILRB2) (56).

Unlike EVs of non-GB origin, GB-EVs uniquely modulate the transcriptome of monocytes, macrophages, and microglia into tumor-supportive phenotypes. Oncogenic EV-uptake by tumor associated cells leads to changes in cytokine secretion (e.g., VEGF and IL-6), changes in antigen display to deviate T cells from their target, and increased expression of matrix metalloproteinases (57) or lowering of miR-146a-5p (58) rendering the extracellular space more permissive for tumor cell migration. Immunosuppression by GB-EVs results in altered release of cytokines from monocytes, such as arginase, TGF-β, and IL-10, which lead to T cell dysfunction in glioma, while blocking pro-inflammatory cytokines such as IL-12, and TNF-α (59). Other functions of GB-EVs include the transfer of information to myeloid cells to obtain pro-tumor immunogenic properties in a process called superinduction (60). Superinduction occurs when exposure of GB cells to IFN-γ during a T-cell response leads to the release of PD-L1 and indoleamine 2,3-dioxygenase (IDO) via GB-derived EVs. These molecules are then internalized by monocytes, promoting the differentiation of myeloid-derived suppressor cells. These myeloid derived suppressor cell, in turn, have the ability to reduce T cell proliferation. Tumor-recruited astrocytes can also contribute to the GB-myeloid immunosuppression circuit. It is known that astrocytes that have taken up GB-derived EVs increase levels of hyaluronic acid (61). This is an important ligand for CD44-positive macrophage differentiation at the GB site attenuating tumor immunogenicity and, consequently, promoting GB growth (62). Other indirect blockage systems involve reducing the functionality of DCs to suppress T-cell maturation, proliferation, and activation (63). Galectin-9 on GB-EVs isolated from the CSF of GB patients binds the TIM-3 receptor on DCs and inhibits antigen recognition, processing, and presentation by DCs and thus a subsequent cytotoxic T cell response (63).

**Figure 3:**
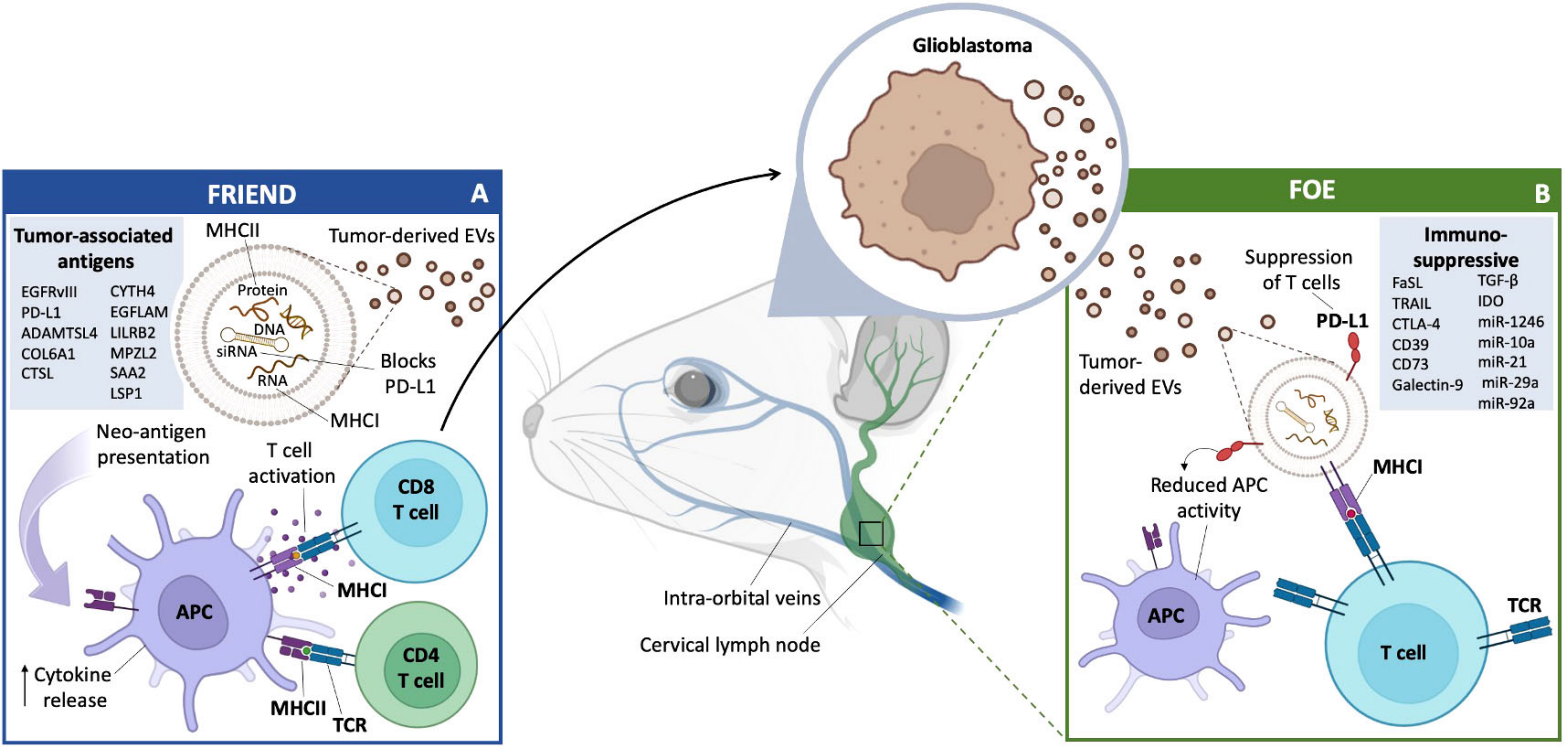
GB EVs and Immunity: Friend or Foe. **(A)** Friend GB-derived EVs travel through the lymphatic system and carry macromolecules from the tumor to various immune system accessory cells, primarily in the cervical lymph nodes. This includes antigen presentation directly to T cells for activation, or T cell activation mediated by APCs and increase in cytokine release. GB-derived EVs can also act as a blockade of PD-L1 secretion, allowing for a more effective immune response. A list of confirmed and potential TAA (left box). **(B)** Foe GB-derived EVs carry immune blocking proteins, such as PD-L1, which can lead to the suppression of T cells and a reduction in APC activity through a number of cytokines, proteins and miRNAs (right box).

In conclusion, EVs derived from a number of cell types in the TME help mediate the immunosuppressive milieu dictated by GB cells (**Figure 3**). These EVs work to ensure that immune cells are not able to respond appropriately to GB neoantigens and thereby aid in tumor establishment and growth, as well as resistance to immunotherapy.

## Friend activity - EVs block tumor progression

### EVs released by TME cells can act to restrict tumor growth/invasion

EVs have emerged as significant regulators of the immune response throughout tumor progression, as they carry a diverse array of molecular cargo that has a critical role in modulating the immune response (64). In recent years, researchers have discovered that brain cell-derived EVs have the potential to suppress tumor growth, offering new insights into the complex interactions between the brain and cancer.

Communication between microglia and GB cells through EVs plays a role in maintaining or restoring the balance of glutamate levels, important in maintaining homeostasis in the central nervous system (CNS) (65). These microglia-derived EVs serve as carriers of specific molecular messages targeted at cancer cells, prompting alterations in the metabolism of GB cells. Notably, these effects are orchestrated by miR-124, encapsulated within small EVs (sEVs) released by microglia which are internalized by GB cells. Once internalized, miR-124 exerts an influence on the behavior and metabolism of GB cells, resulting in a diminished release of lactate, nitric oxide, and glutamate into the extracellular environment. This interplay between microglia and GB cells contributes to the rebalancing of CNS homeostasis and have an effect on how GB cell responses to their surroundings (65). However, it is important to note that the role of microglia secreted EVs in tumor growth can vary depending on the context and specific factors involved.

Another example of EVs acting as a tumor friendly influencer is in regard to oligodendroglioma. These tumor cells release EVs carrying TRAIL and molecular chaperones, which wield their impact by triggering cell demise in astrocytes, potentially hindering tumor growth (66). Moreover, these EVs have the capability to initiate neuronal apoptosis as well (67), which potentially reduces the tumor’s ability to interact with neurons for its benefit an activity against the tumor.

The limited infiltration of NK cells into GB and the effective evasion strategies employed by such tumors have made targeting GB cells challenging (68). However, NK-EVs have been implicated in multiple mechanisms of cancer cell destruction (68, 69) employing both caspase-independent and caspase-dependent pathways to induce cytotoxicity (70). The actions of NK-EVs contribute, at least partially, to the cytotoxic effects observed in NK cell-induced tumor cell death (68, 70). Furthermore, increased levels of specific proteins, such as perforin, granzyme A, granzyme B, and granulysin are associated with the cytotoxic potential of NK-EVs (70).

Endothelial cells in the neovasculature of tumors also put up a good fight. The involvement of endothelial cell-derived EVs carrying esophageal cancer-related gene‐4 (ECRG4) protein can result in the inhibition of glioma cell proliferation (71). Furthermore, the expression of inflammatory cytokines and angiogenesis-related factors, including NF‐κB, IL‐1β, IL‐6, IL‐8, monocyte chemoattractant protein‐1 (MCP‐1), hypoxia‐inducible factor 1‐alpha (HIF‐1α), VEGF and vascular endothelial growth factor receptor 2 (VEGFR2) in the TME are suppressed by ECRG4‐EVs (71). GBs also release EVs containing podoplanin (PDPN), resulting in platelet activation and a clotting cascade, which can lead to thrombosis in the tumor and peripheral vasculature, although definitely a health risk to patients it is not clear whether this helps or hurts the tumor itself.

In conclusion, the release of EVs by different brain cells represents a fascinating avenue in cancer research. The ability of some of these EVs to suppress tumor growth through various mechanisms highlights their potential therapeutic significance. Further exploration of these interactions between brain cells and tumors may help to develop targeted anti-cancer therapies that exploit the natural tumor-suppressive properties of some brain cell-derived EVs.

### EVs released by GBs can travel to cervical lymph nodes to present neo-antigens

An effective T cell response is an important step in mounting an immune response against a tumor. The cervical lymph nodes serve as one of the primary sites of tumor antigen presentation to T cells. DCs, a type of professional antigen presenting cell (APC), expose their loaded antigens to naive T cells, which can prime the T cells into effector T cells, with cytotoxic, regulatory or helper capabilities (72) (**Figure 3**).

To launch an antigen specific response, cytotoxic T-lymphocytes (CTLs) must form a close relationship, called the immune synapse (IS), between themselves and target cells to begin an antigen specific response (73). EVs have been shown to be effective in activating CTLs, by activating naïve T cells, even in the absence of APCs (74). GB is considered to be a “cold” tumor, i.e. low in tumor neoantigens. Neoantigens arise from somatic mutations that occur in coding regions of genes during tumorigenesis and are not found in healthy cells (75).

GB is known for its low mutation burden and a low frequency of mutations in the tumor cells. One of the most recognized neoantigens is EGFRvIII, a mutant form of EGFR found in ~ 30% of GBs (76). Using RNA-seq data from 142 GB patients, 6,585 mutated genes were identified as potential sources of tumor specific antigens (TSAs) and 5,221 genes were overexpressed and identified as potential tumor-associated antigens (TAAs) (77). Of the 1,322 GB-associated genes that fell under both categories, nine genes (ADAMTSL4, COL6A1, CTSL, CYTH4, EGFLAM, LILRB2, MPZL2, SAA2, and LSP1) were identified as being associated with both overall survival and relapse free survival. These nine genes had positive correlations with DC infiltration, implying recognition of potential neoantigens which could be presented to APCs and be involved in an immune response. These potential neoantigens were identified as possible targets for an mRNA vaccine, and may even be transported by GB-derived EVs to elicit an immune response. Tumor cell-derived proteins have been detected in and on EVs isolated from plasma using a cross-species xenograft model, providing additional proof that tumor-derived EVs circulate in the blood (78). As tumor-derived EVs are carriers of neoantigens, it is possible that they could be mediators of an anti-tumor immune response. In two murine glioma models, major histocompatibility complex I (MHC-I) (H-2Db)–restricted Imp3D81N (GL261 cells) and Odc1Q129L (SMA-560 cells) were confirmed to be endogenous neoantigens with immunogenic properties, with neoantigen-specific T cell populations being detected both intratumorally and within cervical lymph nodes (79). Thus, MHCI molecules exposed to tumor antigens on the surface of EVs can directly prime naive T cells into CD8+ T cells with cytotoxic capabilities.

Alternatively, EVs can be taken up by professional APCs, such as DCs, and GB neoantigens presented to T cells as another form of antigen presentation, known as cross-presentation (49). Migratory DCs presenting tumor antigens can travel to regional lymph nodes and present their antigens, or release EVs that will present these antigens to resident-lymphoid DCs through vesicle transfer to facilitate antigen presentation to T cells (49). This process aids circulating naive T cells in becoming active against a corresponding tumor-antigen (49). The process of EVs presenting tumor-antigens to immune cells reveals the potential of EVs to be beneficial in mounting an immune response against GB and other malignancies.

### EVs can serve as therapeutic vehicles

EVs hold promise a means of delivering drugs and other therapeutics to GB in various applications. When administered systemically to rodents, GB EVs have demonstrated the ability to transport functional cargo while evading immune clearance more effectively than conventional delivery methods, such as surgery, chemotherapy and targeted immunotherapy (80), and intravenous administered EVs can pass through the blood-brain barrier (BBB) (81). The latter can be facilitated with focused ultrasound (82).

The therapeutic potential of EVs is further supported by clinical data emerging from cancer research. For comparison nanoparticles (NPs) containing doxorubicin (DOX) were employed for the treatment of intracranial GB (83–85). After uptake by U87 glioma cells, NPs facilitated the release of DOX from lysosomes with cytotoxic effects (84). Red blood cells (RBC) EVs loaded with drugs exhibited no systemic toxicity, while direct doses of DOX demonstrated systemic toxicity at levels which were therapeutically effective. RBC-derived EVs loaded with combination of cytoplasmic phospholipase A2 (cPLA2) siRNA/metformin served to downregulate GB energy metabolism (86). Impaired GB metabolism resulted in reduced tumor growth and increased mouse survival in a patient-derived xenograft GB model (86). miR-1208 loaded EVs led to suppression of the TGB-β pathway and reduction of glioma growth in mice (82). EVs have also been loaded with CRISPR-Cas9 to sensitize glioma cells to radiotherapy by enhancing induction of ferroptosis (87) and with the cytokine IL-12 in EVs from mature DCs to enhance immune response to the tumor (88). These recent examples from the literature illustrate loading of EVs with drugs, RNA and proteins for therapeutic effect on GB.

The specific use of EVs as drug carriers also presents opportunities for immunotherapy. A recent study demonstrated that the CpG-STAT3 antisense oligonucleotides loaded into neural stem cells derived EVs potently stimulated immune activity of human DCs or mouse macrophages, inducing NF-κB signaling and IL-12 production in the glioma microenvironment in mice (89). Furthermore, an anesthetic Propofol suppressed the communication between pro-tumorigenic GB stem cells (GSCs) and microglia by interfering with the transmission of EVs (90). This study substantiated the anticancer attributes of Propofol, delineating its capacity to modulate GSCs, rendering them more receptive to ionizing radiation and temozolomide (TMZ) therapy. Furthermore, Propofol can perturb the pro-tumorigenic interactions between GSCs and microglia when combined with EV-mediated transport of antisense RNA to brain-derived neurotrophic factor (BDNF) (90).

In a rat GB model, EVs carrying yeast cytosine deaminase::uracilphosphoribosyl transferase (yCD::UPRT-MSC) conjugated with 5-fluoroytocysine (5-FC) cured a significant number of rats when injected intraperitoneally or intranasally, with 5-FC being converted to the cytotoxic 5-fluorouracil (91). Recently, it has been shown that adipose stem cell-derived EVs may prove intrinsically therapeutic by regulating, proliferation, invasiveness and angiogenesis of GB cells, as shown in *in vitro* and *in vivo* chorioallantoic membrane model assays (92).

The inherent potential of EVs to selectively interact with target cells upon introduction into an organism is a fundamental characteristic crucial for precise targeting of particular cell populations, such as tumor cells. It has been established that EVs derived from zebrafish brain endothelial cells, carrying paclitaxel and DOX payloads, exhibit the remarkable capability not only to traverse the BBB, but also to exhibit a high degree of specificity in targeting GB cells (93). Other targeting mechanisms for GB have included docking of antibodies to PD-L1 on CD64 on the EV surface for delivery of mRNA for IFN-γ (94) and conjugation of cyclic-RGDyC to the EV surface to target integrin alpha v beta 3 on GB cells to deliver DOX (95). EVs derived from engineered MSCs expressing anti-EGFRvIII antibody on their surface selectively induced apoptosis in U87-EGFRvIII GB cells, as compared to U87 cells *in vitro* (96).

Neoantigens have also proven to be promising candidates for immunotherapy, targeting various malignancies, including GB. In a Phase Ib GB clinical trial, MHCI-based neoantigen vaccines were used to induce an immune response after surgery and chemotherapy (97). The results suggested that levels of infiltrating T cells increased only in patients who developed an immune response specific to the neoepitopes of the vaccine. In a Phase IIa clinical study against newly diagnosed GB, SurVaxM, a peptide vaccine targeting a member of the inhibitor of apoptosis protein family, was shown to be safe with no serious side effects attributed to SurVaxM. A randomized, large-scale clinical trial of SurVaxM is currently ongoing (NCT02455557; source ClinicalTrail.gov) (98). In a Phase I clinical trial, an ITI-1001 multi-antigen DNA vaccine was given to patients with newly diagnosed GB. The ITI-1001 vaccine utilized the UNITE platform, which combines the lysosomal targeting protein LAMP1 with target antigens (pp65, gB, and IE-1) (NCT05698199; source ClinicalTrail.gov). Treatment of a syngeneic GB mouse model (CT-2A) with ITI-1001 resulted in increased antigen presentation, multi-antigen-specific CD4 and CD8 T cell responses, and around 56% long-term survival in tumor-bearing mice (99). These promising findings led to a follow-up phase I clinical trial with GBM patients (NCT05698199; source ClinicalTrail.gov). Further studies could elucidate more potential neoantigens that provide an anti-tumor immune response and apply them to existing EV-nano vaccine approaches targeting GB.

Despite the encouraging prospects of EVs as a prospective diagnostic and therapeutic avenue for GB, there remains a scarcity of clinical-level investigations evaluating their potential therapeutic applications in GB. Although the involvement of EVs in the progression of GB is well-established, substantial challenges persist in harnessing EVs for therapeutic purposes in GB. These challenges encompass the isolation, subtyping, enrichment, cargo loading, and conferring of target specificity to EVs, among other aspects. Overcoming these impediments holds the potential to establish EV-based therapies as a routine treatment modality for glioma patients in the future.

## Informer activity - EVs serve as biomarkers

### EVs released by GBs can serve as biomarkers (informers) for cancer drivers and response to therapy

Currently, magnetic resonance imaging (MRI) is the most commonly used method for detecting GB and monitoring tumor progression. This process is, however, time-consuming, inconvenient for patients, and not always reliable in patients who previously received tumor resection followed by radiotherapy or immunotherapy, referred to as pseudo-progression (100–102). Additionally, invasive tumor biopsies are performed to confirm tumor subtypes and genetic drivers, as well as to tailor treatments precisely to patients (100). Therefore, there is a great need for minimally invasive techniques to detect and monitor GB progression and response to therapy at all stages.

Biomarkers found in blood, CSF, urine or saliva can provide detailed information about tumors using a minimally invasive approach and have a prominent role in tumor diagnosis, and assessment of disease progression and treatment response (102, 103). In GB, however, the search for biomarkers has been challenging mainly due to the restricted permeability of the BBB, which limits the passage of tumor-derived biomarkers into the circulation (102, 104). EVs released by tumor cells and other cells in the TME contain various proteins and RNA species and can pass through the BBB, carrying detailed information about the tumor and the TME, including tumor-specific biomarkers (105). Moreover, EVs have relatively short half-lives in circulation, thereby reporting on the current status of the tumor (106).

EV isolation methods pose many challenges, resulting in either loss of EV quantity or purity of EV samples (107, 108). Nevertheless, new methods are continuously being created to identify EV content, such as Surface-enhanced Raman spectroscopy with nanocavity microchips (MoSERS microchip) (109) and tunable micropattern arrays (110). GB-EVs found in the blood are present in small quantities, especially in early GB stages, and mixed into a complex composition of EVs derived from other cell types, circulating cells, proteins, nucleic acids and lipids (107, 108). Protein biomarkers are found to be valuable biomarkers in a variety of cancers (111, 112), but have shown limitations as biomarkers in GB as serum contains a limited amount of GB-derived protein (107, 111). This makes it challenging to isolate enough tumor-derived protein for early GB diagnostic purposes. As tumor-derive proteins will be present in higher quantities at later stages of tumor progression, proteins could be useful for analyzing treatment response and prognosis. RNA, on the other hand, has great potential as a diagnostic biomarker for GB, as RNA is protected from degradation in EVs and can be amplified after EV isolation (111).

Early studies identified the EGFRvIII mutant RNA in serum of patients harboring EGFRvIII-positive tumors (25). This now includes a host of other tumor markers including amplified EGFR (113); miR-21 (114), miR-486-3p (115); O6-methylguanine DNA methyltransferase, and isocitrate dehydrogenase (116). In patients with GB, the levels of PD-L1 RNA in EVs derived from serum and plasma have exhibited correlation with tumor volume up to 60 cm^3^ (110).

Even though proteins pose challenges as potential biomarkers in early GB diagnosis, several studies have revealed their potential. Cilibrasi et al. (106) compared sEVs (< 200 nm) derived from the plasma of healthy controls with those from GB patients, explicitly looking at protein content within EVs. They found ninety-four proteins derived from EVs to be significantly different between GB patients and healthy controls, including Von Willebrand-Factor (VWF), complement signature C3, Fc Gamma Binding Protein (FCGBP), Protein S 1 (PROS1) and Serpin Family A Member 1 (SERPINA1). These proteins were previously identified as linked to GB and have been associated with immune evasion and poor prognosis (106). In addition, CD29, CD44, CD146, CD81, C1Qa, and histone H3 were also recently identified as potential protein markers for the tumor progression of GB. These markers were upregulated in sEVs of recurrent GB patients and are associated with angiogenesis, invasiveness, and proliferation (102). Another protein that promotes angiogenesis, LGALS3BP is overexpressed in plasma EVs of several cancers, including GB, making it a good marker for GB diagnosis (117). The EV levels of LGALS3BP are thought to correlate with tumor grade and increased tumor burden (117, 118).

Saliva also contains EVs with protein biomarkers (105). Some potential biomarkers found in sEVs of saliva are aldolase A (ALDOA), 14‐3‐3 protein ϵ (1433E), transmembrane protease serine 11B (TM11B), and enoyl CoA hydratase 1 (ECH1). These proteins are increasingly present in pre-operative GB patients with unfavorable outcomes compared to pre-operative patients with favorable outcomes. These proteins have a role in cell proliferation, apoptosis, migration, and invasion, potentially increasing the likelihood of poor outcomes in patients (105).

Research studies vary with regard to EV size and concentration in GB patients compared to healthy controls. The majority of studies indicate higher EV levels in patients compared to healthy subjects (3, 102, 103). Whereas others, found no significant differences (106). The variation in findings among research groups might be attributed to differences in EV isolation techniques or patient cohorts (106, 119).

In addition to sEVs derived from plasma or saliva, sEVs isolated from the CSF may also provide in-depth information about the internal interactions between the tumor and the TME. As these sEVs will not have passed through the BBB, they may provide a more accurate representation of the current situation within the TME. EVs isolated from CSF have shown higher sensitivity in detecting GB than EVs isolated from other bodily fluids (104, 120). This method of EV isolation, however, is more invasive for patients, making it a more challenging method for tumor monitoring and treatment response (5, 120). An example of CSF biomarkers released in EVs of GB cells and glioma stem cells are miR-21 and miR-9 (120). These potential biomarkers play a role in tumor migration and proliferation.

Recently, urine samples of GB patients have been studied and urine-derived EVs contain useful diagnostic and prognostic biomarkers for GB (119, 121). It is challenging to extract EVs and biomarkers from urine, but several research groups have shown that nanowire assays can be a useful tool for extracting these biomarkers. Urine is easily accessible and would provide a minimally invasive way to diagnose or analyze treatment response in GB. Fifty-seven mi-RNAs are differentially expressed in patients with CNS tumors compared to healthy subjects, of which 23 most strongly associated with GB were selected using logistic LASSO regression analysis - miR-6070, miR-22-3p, miR-4538, miR-1285-3p, miR-372-5p, miR-4525, miR-5698 were increasingly expressed in CNS tumor patients, including GB patients, and miR-204-3p, miR-6763-5p, miR-101-5p, miR-208a-5p, miR-371a-3p, miR-378a-5p, miR-216a-5p, miR-6864-3p, miR-450b-3p, miR-640, miR-4426, miR-17-3p, miR-450a-2-3p, miR-1248, miR-100-5p, and miR-16-5p were under expressed in these patients as compared to controls (121). In addition, the EV membrane protein CD31/CD63 was overexpressed in urine of GB patients compared to healthy patients (119). CD31 is correlated with tumor progression and prognosis as it plays a role in vasculogenesis (119). All in all, urine has potential as a source for EV biomarkers, but further research needs to explore the extent and application of EV biomarkers present in urine, as well as how small the EVs need to be to pass through the kidney filtration. Besides using EVs in GB diagnosis, EVs can serve as predictive markers for treatment response and can, thus, have a role in personalized medicine. Several studies observed increased expression of heat shock proteins (HSPs), mainly HSP70, in both sEVs (< 200 nm) and the TME of TMZ resistant human glioma lines tested *in vitro*, most prominently U87 (122–124). TMZ-sensitive U87 cells showed a decrease in HSPs compared to untreated U87 cells (123). HSPs are proteins broadly involved in proteome homeostasis, with HSP70 specifically playing a role in treatment resistance and increasing survival in cultured U87 cells (123). Similar decreases of isocitrate dehydrogenase type 1 (IDH1), PDPN, and Hsp90 were observed in both sEVs (< 200 nm) and large (l) EVs (> 200 nm) of TMZ treatment-sensitive GB cells in culture compared to untreated samples. HSPs, IDH1 and PDPN have great potential as predictive markers for GB and should be further explored *in vivo*.

RNA content of EVs is known to be a highly sensitive and predictive biomarker of GB. Upregulation in the long-non-coding RNA - SBF2-AS1 was associated with TMZ resistance *in vitro* and *in vivo* (122). Moreover, Dacomitinib, a tyrosine kinase inhibitor, showed differences in gene expression in recurrent GB with amplified EGFR as measured in EV mRNA comparing GB patients with responsive and non-responsive tumors. EGFRvIII or EGFR-extracellular domain (ECD) mutation status was, however, not correlated with the clinical response (122, 125). In addition, expression of the *RAD51* gene and the *MDM2* gene increase significantly in the TME of the U87 MG and LN229 glioma cell lines in association with HSP proteins *in vitro*. MDM2 plays a role in the degradation of p53, resulting in evasion of apoptosis. An increase of MDM2 is observed in both the TME and EVs, whereas RAD51 is only increased in the TME (124). RAD51 is involved in DNA repair mechanisms, so increasing expression of this gene could fortify resistance in treatment that induces DNA damage, such as chemotherapy. In clinical tissue samples and serum derived-EVs from GB patients, the lncRNA HOX transcript antisense RNA (HOTAIR) was overexpressed, which has great potential as a prognostic and diagnostic biomarker and may also provide further information about TMZ treatment resistance (126).

Ding et al. (127) compared GB patients’ immunological states and treatment responses and categorized patients based on EV gene expression and survival (high risk vs. low risk). Three EV transcripts for nerve growth factor (NGF), insulin‐like growth factor binding protein 6 (IGFBP6), and T cell receptor constant β chain-1 (TRBC1) were identified as the most relevant features in predicting patients’ risk. The high-risk group showed significantly shorter survival compared to low-risk group. In addition, they demonstrated differences in immune cell invasion, with the high-risk groups expressing more immune checkpoint markers, including programmed cell death protein (PD1), and having a worse prognosis. Due to the expression of PD1/PD-L1, these patients were found to have improved responses when treated with anti-PD-L1 compared to those in the low-risk group. On the other hand, the low-risk patients displayed more somatic mutations, such as in EGFR and TP53. Targeted therapies may, thus, benefit patients in the low-risk groups. Therefore, exploring gene expression in EVs from patients may be valuable for anticipating treatment response and might aid in the development of personalized therapies for patients (122, 125, 127).

Finally, positive treatment response to photon- and proton-based radiotherapy showed an increase in patients with EVs expressing the cell surface proteins - CD9 and CD81 (128). A substantial increase in CD9- and CD81-positive EVs post-treatment was found to be a valuable indicator of treatment effect. This increase was also observed when GB cells (A172, LN229, U373, and T98G) in culture underwent apoptosis. An increased presence of CD9- and CD81-positive EVs are not, however, indicative of radiosensitivity, as these markers are highly expressed in the majority of GB patient EVs regardless of how effective the treatment is (128).

**Figure 4:**
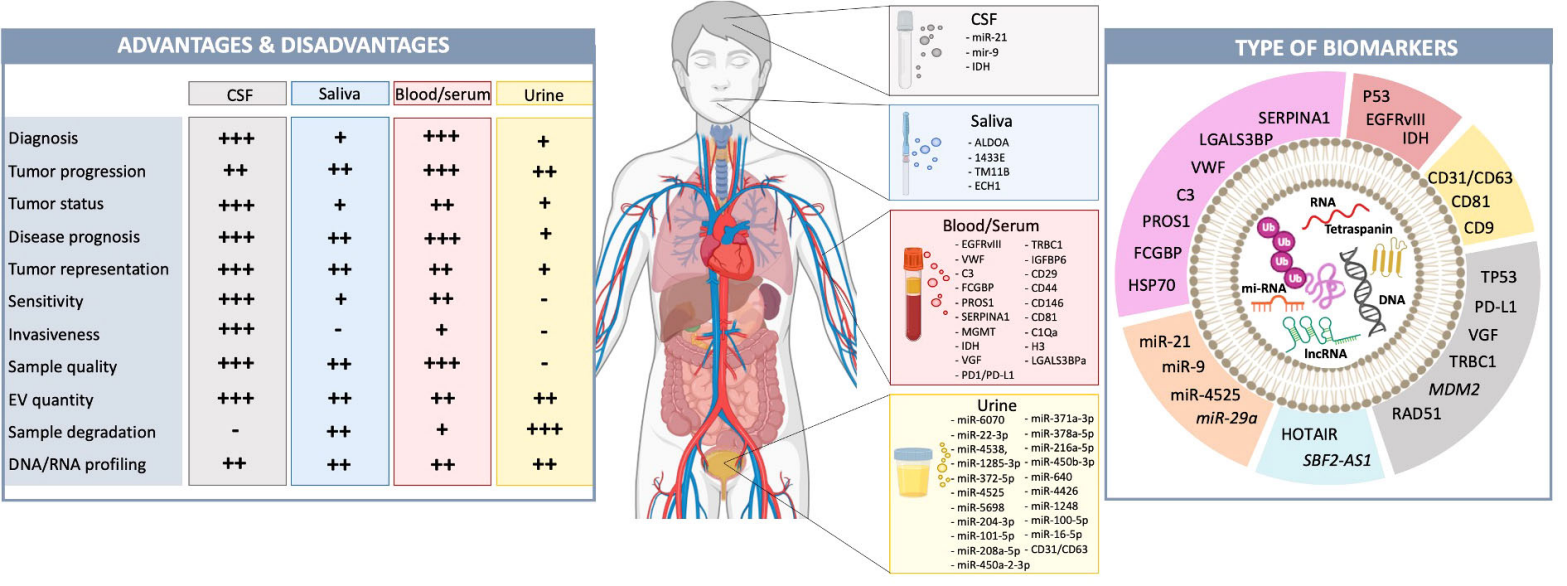
GB-derived EVs function as biomarkers in liquid biopsies. Liquid biopsies from blood, CSF, urine, or saliva are becoming valuable, minimally invasive tools for tumor diagnosis and prognosis (102, 103). Each type has advantages and disadvantages (left). CSF is closest to the tumor and in contact with the TME, accurately representing the current tumor status (102, 103). However, obtaining CSF is highly invasive, making it more challenging to analyze tumor progression and treatment response. Biomarkers in saliva, blood and urine have crossed the BBB and have, thus, been filtered. This is even more extreme in urine as they travel through the kidneys (129, 130). This makes these liquid biopsies less reliable for analyzing tumor status and representation, however, obtaining these biopsies is less invasive for patients. Previously identified potential biomarkers have been listed. The source (CSF, saliva, blood/serum, urine) and type (i.e., RNA, DNA, protein, mi-RNAs). of these EV biomarkers are illustrated (pink, protein; red, RNA; yellow, tetraspanin; grey, DNA; blue, lon-noncoding RNA; orange, mi-RNA) (right). Biomarkers written in italics are identified in pre-clinical models, but not in patient samples yet.

In conclusion, EVs can be valuable biomarkers for GB diagnosis, tumor progression and treatment response (**Figure 4**). EV size and concentration are currently unreliable for GB diagnosis or progression, but protein and RNA markers can be informative. Further research is needed to expand distinguishing EV characteristics in GB patients of various types, at different stages of the disease and in response to therapy.

## Conclusion

EVs function as cargo vehicles transporting a variety of cellular contents throughout the TME and are therefore promising as biomarkers and even targets for therapeutic approaches in treatment of cancer, including GB. However, EVs – especially those released from tumor cells are very adept at promoting tumor progression, including changing the phenotype of normal cells in the TME so that they come to support tumor growth. In addition these EV enemies of the brain have multiple roles in suppressing the immune response to the tumor. All in all it is sometimes difficult to categorize EVs as friends or foes as they have so many cross acting communicative functions. In general, early in tumor growth EVs from normal cells, such as microglia and astrocytes, are focused on eliminating this “foreign object”, but later as the tumor progresses and bombards them with its own EVs they switch camps and are more supportive of the tumor. In this review we have summarized the roles of EVs in protection of brain from GB and tumor progression and discussed recent and current research regarding the use of EVs as a diagnostic and therapeutic tool.

## Author contributions

TRL: Writing – original draft, Writing – review & editing. LN: Visualization, Writing – original draft, Writing – review & editing. ABV: Writing – original draft. AZ: Writing – original draft. YS: Writing – original draft. KB: Writing – original draft, Writing – review & editing. XOB: Funding acquisition, Supervision, Writing – original draft, Writing – review & editing.
