# Supplementary figures and images for: Roles of extracellular vesicles in glioblastoma: foes, friends and informers
Source: Front Oncol. 2023 Nov 24;13:1291177. doi: 10.3389/fonc.2023.1291177 (PMC10704464; doi:10.3389/fonc.2023.1291177)

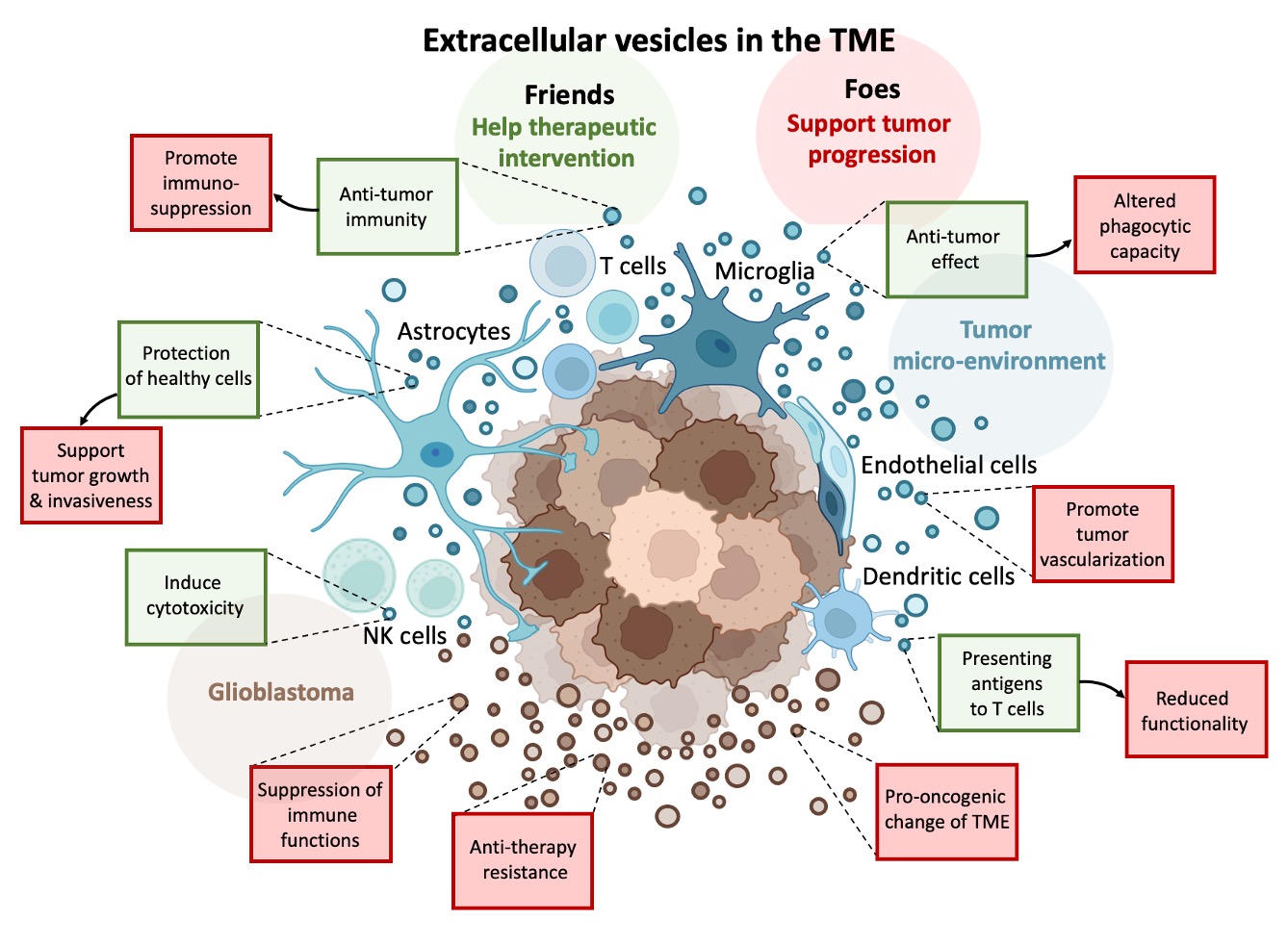

Supplement: Supplementary file 1 [file Image_1.jpeg]
